# Triglyceride‐glucose index and clinical outcomes in sepsis: A retrospective cohort study of MIMIC‐IV

**DOI:** 10.1111/jcmm.70007

**Published:** 2024-08-28

**Authors:** Yan Cao, Liudang He, Yingjie Su, Ju Luo, Ning Ding

**Affiliations:** ^1^ Department of Emergency Medicine Hunan Provincial People's Hospital (The First Affiliated Hospital of Hunan Normal University) Changsha China; ^2^ Department of Emergency Medicine, The Affiliated Changsha Central Hospital, Hengyang Medical School University of South China Changsha China; ^3^ Department of Geriatrics, The Affiliated Changsha Central Hospital, Hengyang Medical School University of South China Changsha China

**Keywords:** MIMIC‐IV, mortality, sepsis, triglyceride‐glucose index

## Abstract

Although accumulating researches were done for investigating the relationship between triglyceride‐glucose index (TyG index) and different diseases, none of the researches have been made in sepsis yet. In this study, we aimed to explore the relationship between TyG index and clinical outcomes in sepsis based on a large critical care public database. Sepsis patients in Medical Information Mart for Intensive Care IV (MIMIC‐IV) Database were included. The exposure was TyG index, which was calculated by the equation: ln (TG (mg/dL) × FBG (mg/dL)/2). The outcomes were in‐hospital mortality and 1‐year mortality. The relationship between TyG index and outcomes was performed by Cox regression analysis. Smooth fitting curves were constructed by using generalized additive model. Kaplan–Meier analyses for cumulative hazard of 1‐year mortality in different groups were done. 1103 sepsis patients were included with a median TyG index of 9.78. The mortalities of in‐hospital and 1‐year were 37.53% (*n* = 414) and 42.25% (*n* = 466), respectively. After adjusting confounders, there was a significantly negative relationship between TyG index and mortalities of in‐hospital and 1‐year. With the per unit increment in TyG index, the risk of in‐hospital and 1‐year mortality both decreased by 21% (HR = 0.79, 95% CI: 0.66–0.94, *p* = 0.0086 and HR = 0.79, 95% CI: 0.66–0.94, *p* = 0.0080, respectively). A negative relationship between TyG index and clinical outcomes in sepsis was found.

## 1 INTRODUCTION

The triglyceride‐glucose index (TyG index), which is defined on the basis of calculating the values of triglycerides (TG) and fasting blood glucose (FBG) has been identified as prognostic indicators in various disorders including metabolic disease,^1^ cardiovascular disease,^2^ digestive disease, malignant tumour^3^ and even COVID‐19.^4^

Most of the studies showed the positive relationship between TyG index and clinical outcomes. A study including 1618 patients with coronary heart disease admitted in the intensive care unit (ICU) found that as TyG index increasing, the risk of hospital mortality and ICU mortality both increased.^5^ One recent meta‐analysis study enrolled 18 different researches with a total of 592,635 patients demonstrated that the elevated levels of TyG index were linked with elevated risk of ischemic stroke. Moreover, the ischemic stroke patients with higher levels of TyG index had poorer outcomes.^6^ In patients who had chronic kidney diseases and coronary artery diseases, the relationship between TyG index and the risk of 1‐year mortality was significantly positive (hazard ratio (HR) =1.343, *p* < 0.05).^7^ However, some other researches found there might be a U‐shape relationship between TyG index and some diseases. A public database research included 9254 middle age and elderly participants and illuminated that those participants with the third quartile level of TyG index had the minimal risk of all‐cause mortality.^8^ One longitudinal analysis including 11,851 participants with a median follow‐up of 24.26 years in China concluded that there was a U‐shape association between TyG index and the morbidity of atrial fibrillation.^9^ In the US participants, the relationship between TyG index and the morbidity of diabetic retinopathy was also the U‐shape.^10^

However, none of the researches have been made in sepsis yet. In this study, we aimed to discuss the possible relationship between TyG index and outcomes of short‐term and long‐term in sepsis based on a large critical care public database.

## 2 METHODS

### 2.1 Database and patients

We conducted this retrospective cohort study based on a US public database: Medical Information Mart for Intensive Care IV (MIMIC‐IV) Database.^11^, ^12^, ^13^ All sepsis patients with sepsis 3.0 criteria were included.^14^ Exclusion criteria were as follows: (1) Without records of FBG and TG in 24 h after admission; (2) Age less than 18‐year‐old; (3) Diabetes and patients with antidiabetic treatment (insulin or oral antidiabetics); (4) Died within 48 h after admission; (5) Patients with acute pancreatitis; (6) Patients with dyslipidemia and patients with receiving lipid‐lowering drugs.

Flow chart was demonstrated in Figure S1. Initially, sepsis patients with both FBG and TG levels in 24 h after admission were included (*n* = 1767). After excluding some patients, 1103 sepsis patients were included in the final cohort.

### 2.2 Exposure and outcomes

The exposure variable was the TyG index (TyG index = ln (TG (mg/dL) × FBG (mg/dL)/2)). The main outcomes were in‐hospital mortality and 1‐year mortality.

### 2.3 Variables and parameters

Baseline characteristics were included within 24 h after admission as follows: age, gender, marital status, ethnicity, managements and therapies including renal replacement therapy (RRT), ventilator use and vasopressor use, organ dysfunction including septic shock and acute kidney function (AKI), length of stay (LOS) in ICU and hospital, disease severity scores, comorbidities, vital signs and laboratory parameters.

### 2.4 Statistical analysis

We used and the packages R (http://www.R‐project.org) software and the EmpowerStats (http://www.empowerstats.com) software for performing statistical analysis. Statistical significance was confirmed while there was a two‐sides *p* < 0.05.

First, based on the in‐hospital mortality, the cohort was divided into survivor group and non‐survivor group. Second, based on the tertiles of TyG index, the cohort was divided into three different groups (T1: <9.47, T2: 9.47–10.16, T3: >10.16). General characteristics were compared. Continuous and categorical parameters were indicated as the median with interquartile ranges and the percentages, respectively. The methods of Mann–Whitney *U*‐test or chi‐squared test were applied. Third, Cox regression analysis was performed to investigate the relationship between TyG index and prognosis in sepsis patients. We constructed three different models including Crude model (adjusting for none), Model A (adjusting for age and gender) and Model B (a fully adjusted model). TyG index was investigated not only as a continuous variable but also as a categorial variable (tertiles: T1‐–T3; quartiles: Q1–Q4 (Q1: <9.27, Q2: 9.27–9.77, Q3: 9.78–10.39, Q4: >10.39)). Fourth, Model I (linear model) and Model II (non‐linear model) were compared. If the *p* value <0.05, model II was the better. Otherwise, Model I was better. Fifth, smooth fitting curves were performed for showing the relationship between TyG index and prognosis by using generalized additive model. In addition, Kaplan–Meier analyses were made for cumulative hazard of 1‐year mortality in sepsis patients in different groups (T1–T3, Q1–Q4). Finally, we also did the subgroups analysis for figuring out the stability of our results.

## 3 RESULTS

### 3.1 General characteristics of the study cohort

In this study, 1103 sepsis patients were included based on inclusion and exclusion criteria. The median age was 61‐years‐old, 58.20% were males, 42.16% were married and 64.55% were White (Table S1). The proportions of RRT, ventilation use and vasopressor use during hospitalization were 23.12%, 80.96% and 32.18%, respectively. The incidences of septic shock and AKI during hospitalization were 44.70% and 77.33%, respectively. The mortalities of in‐hospital and 1‐year were 37.53% (*n* = 414) and 42.25% (*n* = 466), respectively.

### 3.2 Comparison of baseline characteristics between in‐hospital survivor group and non‐survivor group

Table 1 showed the different variables in the study cohort. The median values of FBG and TG were 126 mg/dL and 137 mg/dL, respectively. The median TyG index was 9.78. The median days of LOS in ICU and hospital were 11.29 and 20.95, respectively. Clinical characteristics between survivor group (*n* = 689) and non‐survivor group (*n* = 414) based on in‐hospital mortality were compared. Variables including age (*p* = 0.005), TyG index (*p* = 0.002), total bilirubin (*p* < 0.001), total calcium (*p* < 0.001), chloride (*p* = 0.035), haematocrit (*p* < 0.001), haemoglobin (*p* < 0.001), INR (*p* = 0.005), lactate (*p* = 0.020), PLT (*p* < 0.001), PT (*p* = 0.003), PTT (*p* = 0.005), RDW (*p* < 0.001) and RBC (*p* < 0.001) showed significant differences between two groups. The incidences of AKI (*p* < 0.001), RRT (*p* < 0.001) and vasopressor use (*p* < 0.001) were significantly higher in non‐survivor group.

**TABLE 1:** Clinical characteristics between survivor group and non‐survivor group based on in‐hospital mortality.

Variables	Total	Survivor group	Non‐survivor group	*p*‐value
Number	1103	689	414	
Age (years) (median, IQR)	61.00 (50.00–71.00)	60.00 (48.00–69.00)	63.00 (53.00–72.00)	0.005
Gender (*n*, %)				
Male	642 (58.20%)	401 (58.20%)	241 (58.21%)	0.997
Female	461 (41.80%)	288 (41.80%)	173 (41.79%)	
Comorbidities (*n*, %)				
Hypertension	194 (17.59%)	123 (17.85%)	71 (17.15%)	0.767
Renal disease	35 (3.17%)	20 (2.90%)	15 (3.62%)	0.509
CAD	66 (5.98%)	37 (5.37%)	29 (7.00%)	0.268
Variables (median, IQR)				
SBP (mmHg)	112.00 (99.00–128.00)	112.00 (97.00–128.00)	111.50 (100.00–125.00)	0.698
DBP (mmHg)	65.00 (55.00–76.00)	64.00 (55.00–75.00)	65.00 (55.00–77.00)	0.776
HR (beats/min)	98.00 (84.00–114.00)	98.00 (83.00–114.00)	98.00 (85.00–113.00)	0.887
RR (beats/min)	21.00 (17.00–25.00)	21.00 (17.00–25.00)	21.00 (17.00–25.00)	0.265
TyG index	9.78 (9.28–10.39)	9.84 (9.36–10.43)	9.68 (9.16–10.26)	0.002
ALT (IU/L)	30.00 (17.00–65.50)	31.00 (16.00–65.00)	29.00 (18.00–70.75)	0.529
AG (mmol/L)	15.00 (13.00–18.00)	15.00 (13.00–18.00)	16.00 (13.00–19.00)	0.304
Albumin (g/dL)	2.70 (2.30–3.10)	2.70 (2.30–3.10)	2.70 (2.20–3.10)	0.667
AST (IU/L)	47.00 (25.00–102.00)	45.00 (24.00–92.00)	50.00 (26.00–121.00)	0.572
Bicarbonate (mmol/L)	22.00 (18.00–25.00)	21.00 (18.00–25.00)	22.00 (18.00–25.00)	0.475
Total bilirubin (mg/dL)	0.80 (0.40–2.30)	0.70 (0.40–1.80)	1.00 (0.50–3.52)	<0.001
Total calcium (mg/dL)	8.00 (7.40–8.60)	7.90 (7.30–8.60)	8.20 (7.50–8.70)	<0.001
Chloride (mmol/L)	103.00 (98.00–107.00)	103.00 (99.00–108.00)	102.00 (97.00–106.75)	0.035
Creatinine (mg/dL)	1.20 (0.80–2.10)	1.20 (0.80–2.00)	1.30 (0.90–2.40)	0.733
FBG (mg/dL)	126.00 (100.00–163.00)	127.00 (103.00–162.00)	124.50 (96.00–164.00)	0.137
Haematocrit (%)	32.10 (27.00–36.80)	32.60 (28.10–37.30)	30.60 (25.60–36.10)	<0.001
Haemoglobin (g/dL)	10.45 (8.80–12.10)	10.70 (9.00–12.30)	10.00 (8.30–11.80)	<0.001
INR	1.40 (1.20–1.80)	1.40 (1.20–1.70)	1.40 (1.20–2.00)	0.005
Lactate (mmol/L)	2.00 (1.30–3.10)	2.00 (1.30–3.00)	2.10 (1.50–3.30)	0.020
PLT (×10^9^/L)	177.00 (110.25–274.00)	191.00 (123.00–291.00)	162.00 (93.00–251.00)	<0.001
PT (s)	15.10 (13.30–19.40)	15.00 (13.30–18.00)	15.50 (13.20–21.67)	0.003
PTT (s)	32.80 (28.40–40.10)	32.20 (28.10–37.80)	34.15 (29.22–45.00)	0.005
RDW (%)	15.40 (14.10–17.30)	15.10 (13.80–16.70)	16.20 (14.50–18.50)	<0.001
RBC (×10^12^/L)	3.47 (2.92–4.05)	3.58 (3.05–4.11)	3.26 (2.75–3.88)	<0.001
Sodium (mmol/L)	137.50 (134.00–141.00)	138.00 (134.00–141.00)	137.00 (133.00–141.00)	0.281
TG (mg/dL)	137.00 (90.50–218.00)	144.00 (96.00–227.00)	127.50 (81.25–202.75)	0.292
Urea nitrogen (mg/dL)	25.00 (16.00–47.00)	24.00 (16.00–45.00)	28.00 (17.00–50.00)	0.073
WBC (×10^9^/L)	11.60 (7.10–17.30)	11.80 (7.30–17.00)	11.40 (6.80–17.40)	0.834
Organ dysfunction (*n*, %)				
Septic shock	493 (44.70%)	305 (44.27%)	188 (45.41%)	0.711
AKI	853 (77.33%)	508 (73.73%)	345 (83.33%)	<0.001
Managements (*n*, %)				
RRT	255 (23.12%)	126 (18.29%)	129 (31.16%)	<0.001
Ventilator use	893 (80.96%)	551 (79.97%)	342 (82.61%)	0.280
Vasopressor use	355 (32.18%)	185 (26.85%)	170 (41.06%)	<0.001
Scoring systems (IQR)				
APACHEII	11.00 (8.00–14.00)	11.00 (8.00–14.00)	12.00 (9.00–15.00)	<0.001
SOFA	3.00 (2.00–5.00)	2.00 (2.00–4.00)	3.00 (2.00–5.00)	<0.001
Clinical outcomes (days)				
LOS in ICU	11.29 (5.97–19.14)	10.64 (5.56–18.98)	12.20 (6.99–19.64)	0.013
LOS in hospital	20.95 (12.83–33.27)	22.82 (14.69–35.82)	17.81 (10.24–30.46)	0.004

Abbreviations: AG, anion gap; AKI, acute kidney injury; ALT, alanine aminotransferase; APACHE, acute physiology and chronic health evaluation; AST, aspartate aminotransferase; CAD, coronary artery disease; DBP, diastolic blood pressure; FBG, fasting blood sugar; HR, heart rate; ICU, intensive care unit; INR, international normalized ratio; IQR, interquartile ranges; LOS, length of stay; PLT, platelet; PT, prothrombin time; PTT, partial thrombin time; RBC, red blood cells; RDW, red blood cell distribution width; RR, respiratory rate; RRT, renal replacement therapy; SBP, systolic blood pressure; SOFA, sequential organ failure assessment; TG, triglyceride; TyG index, triglyceride glucose index; WBC, white blood cells.

### 3.3 Comparison of baseline characteristics between three groups based on tertiles of TyG index

Based on tertiles of TyG index, we divided the cohort into three groups: T1 (<9.47, *n* = 368), T2 (9.47–10.16, *n* = 367), T3 (>10.16, *n* = 368) (Table 2). The mortalities of in‐hospital in T1–T3 groups 42.12%, 38.42% and 32.07%, respectively (*p* = 0.017). The mortalities of 1‐year in T1–T3 groups 47.28%, 42.51% and 36.96%, respectively (*p* = 0.018). Significant differences were showed in age (*p* < 0.001), HR (*p* = 0.002), RR (*p* < 0.001), ALT (*p* = 0.039), AG (*p* = 0.009), albumin (*p* = 0.027), total bilirubin (*p* = 0.034), total calcium (*p* = 0.007), haematocrit (*p* = 0.042), haemoglobin (*p* = 0.017), FBG (*p* < 0.001), INR (*p* = 0.002), PT (*p* = 0.002), PTT (*p* = 0.004), RDW (*p* < 0.001), RBC (*p* < 0.001), TG (*p* < 0.001), RRT (*p* = 0.036), and LOS in hospital (*p* = 0.049).

**TABLE 2:** Clinical characteristics between three groups based on tertiles of TyG index.

TyG index (tertiles)				
Variables	T1 (<9.47)	T2 (9.47–10.16)	T3 (>10.16)	*p*‐value
Number	368	367	368	
Age(years) (median, IQR)	64.00 (52.00–73.00)	61.00 (51.00–70.00)	59.00 (46.00–68.50)	<0.001
Gender (*n*, %)				
Male	218 (59.24%)	211 (57.49%)	213 (57.88%)	0.881
Female	150 (40.76%)	156 (42.51%)	155 (42.12%)	
Comorbidities (*n*, %)				
Hypertension	72 (19.57%)	60 (16.35%)	62 (16.85%)	0.468
Renal disease	14 (3.80%)	11 (3.00%)	10 (2.72%)	0.683
CAD	24 (6.52%)	19 (5.18%)	23 (6.25%)	0.719
Variables (median, IQR)				
SBP (mmHg)	111.00 (98.00–123.00)	112.00 (100.00–129.00)	113.00 (99.00–131.00)	0.364
DBP (mmHg)	62.50 (53.00–76.00)	65.00 (55.00–77.00)	65.00 (56.00–74.00)	0.433
HR (beats/min)	94.00 (81.00–111.00)	98.00 (85.00–112.00)	102.50 (88.00–118.00)	0.002
RR (beats/min)	20.00 (16.00–24.00)	21.00 (17.00–25.00)	22.00 (18.00–26.00)	<0.001
TyG index	9.05 (8.79–9.28)	9.78 (9.64–9.97)	10.70 (10.39–11.16)	<0.001
ALT (IU/L)	26.00 (15.00–52.50)	30.00 (16.00–71.50)	34.00 (20.00–75.25)	0.039
AG (mmol/L)	15.00 (12.00–18.00)	15.00 (13.00–18.00)	16.00 (13.00–19.00)	0.009
Albumin (g/dL)	2.70 (2.30–3.20)	2.70 (2.30–3.10)	2.60 (2.20–3.00)	0.027
AST (IU/L)	45.00 (24.00–88.00)	47.00 (24.00–108.00)	50.50 (27.00–107.75)	0.147
Bicarbonate (mmol/L)	21.50 (18.00–25.00)	22.00 (19.00–25.00)	21.00 (18.00–25.00)	0.063
Total bilirubin (mg/dL)	0.90 (0.50–3.00)	0.70 (0.40–2.00)	0.80 (0.40–2.00)	0.034
Total calcium (mg/dL)	8.10 (7.50–8.70)	8.10 (7.50–8.70)	7.90 (7.20–8.50)	0.007
Chloride (mmol/L)	104.00 (99.00–109.00)	103.00 (98.00–107.00)	102.00 (98.00–107.00)	0.051
Creatinine (mg/dL)	1.20 (0.80–2.02)	1.20 (0.80–2.10)	1.30 (0.80–2.40)	0.095
FBG (mg/dL)	103.00 (84.00–125.25)	125.00 (104.00–154.00)	162.50 (128.75–231.00)	<0.001
Haematocrit (%)	31.40 (26.60–35.80)	31.90 (26.55–36.85)	32.70 (27.60–37.50)	0.042
Haemoglobin (g/dL)	10.20 (8.70–11.75)	10.30 (8.70–12.20)	10.70 (8.90–12.30)	0.017
INR	1.50 (1.20–2.00)	1.40 (1.20–1.80)	1.30 (1.20–1.60)	0.002
Lactate (mmol/L)	2.00 (1.30–3.12)	2.00 (1.30–3.00)	2.20 (1.40–3.30)	0.104
PLT (×10^9^/L)	173.00 (108.00–261.00)	185.00 (115.00–290.00)	176.00 (111.00–270.00)	0.083
PT (s)	16.00 (13.40–21.30)	15.10 (13.30–19.70)	14.70 (13.00–17.58)	0.002
PTT (s)	35.60 (30.40–44.30)	32.85 (28.70–39.38)	30.85 (27.18–36.42)	0.004
RDW (%)	15.80 (14.20–17.90)	15.30 (14.20–17.30)	15.00 (13.80–16.92)	<0.001
RBC (×10^12^/L)	3.36 (2.86–3.88)	3.44 (2.88–4.10)	3.62 (3.05–4.15)	<0.001
Sodium (mmol/L)	138.00 (134.00–142.00)	137.00 (134.00–140.50)	137.00 (134.00–141.00)	0.923
TG (mg/dL)	79.00 (61.75–102.00)	140.00 (111.50–174.00)	268.00 (189.75–405.25)	<0.001
Urea nitrogen (mg/dL)	25.00 (16.00–45.00)	24.00 (15.00–45.50)	27.00 (17.00–49.25)	0.246
WBC (×10^9^/L)	10.80 (6.60–16.70)	11.50 (7.00–16.80)	12.60 (8.10–18.33)	0.360
Organ dysfunction (*n*, %)				
Septic shock	149 (40.49%)	166 (45.23%)	178 (48.37%)	0.096
AKI	280 (76.09%)	81 (77.93%)	287 (77.99%)	0.605
Managements (*n*, %)				
RRT	69 (18.75%)	88 (23.98%)	98 (26.63%)	0.036
Ventilator use	287 (77.99%)	302 (82.29%)	304 (82.61%)	0.204
Vasopressor use	115 (31.25%)	121 (32.97%)	119 (32.34%)	0.880
Scoring systems (IQR)				
APACHEII	11.00 (8.00–15.00)	11.00 (9.00–14.00)	11.00 (8.00–14.00)	0.965
SOFA	3.00 (2.00–5.00)	2.00 (2.00–4.00)	3.00 (2.00–5.00)	0.428
Clinical outcomes (days)				
LOS in ICU	10.01 (5.53–17.59)	12.09 (5.77–21.00)	11.74 (6.81–19.67)	0.131
LOS in hospital	20.70 (11.87–32.55)	22.32 (14.23–37.88)	20.42 (11.91–31.18)	0.049
In‐hospital mortality (*n*, %)	155 (42.12%)	141 (38.42%)	118 (32.07%)	0.017
1‐year mortality (*n*, %)	174 (47.28%)	156 (42.51%)	136 (36.96%)	0.018

Abbreviations: AG, anion gap; AKI, acute kidney injury; ALT, alanine aminotransferase; APACHE, acute physiology and chronic health evaluation; AST, aspartate aminotransferase; CAD, coronary artery disease; DBP, diastolic blood pressure; FBG, fasting blood sugar; HR, heart rate; ICU, intensive care unit; INR, international normalized ratio; IQR, interquartile ranges; OS, length of stay; PLT, platelet; PT, prothrombin time; PTT, partial thrombin time; RBC, red blood cells; RDW, red blood cell distribution width; RR, respiratory rate; RRT, renal replacement therapy; SBP, systolic blood pressure; SOFA, sequential organ failure assessment; TG, triglyceride; TyG index, triglyceride glucose index; WBC, white blood cells.

### 3.4 The relationship between TyG index and mortalities of in‐hospital and 1‐year in crude and adjusted models

Table 3 demonstrated that in adjusted model B (fully adjusted model), there was a negative relationship between TyG index and mortalities of in‐hospital and 1‐year. The HRs were 0.79 (95% CI: 0.66–0.94, *p* = 0.0086) and 0.79 (95% CI: 0.66–0.94, *p* = 0.0080), respectively. Compared to T1 group (TyG index<9.47), the risk of mortalities of in‐hospital and 1‐year in T3 group (TyG index>10.16) were significantly lowest (HR = 0.67 (95% CI: 0.46–0.96, *p* = 0.0314; *p* for trend 0.0315) and HR = 0.66 (95% CI: 0.46–0.95, *p* = 0.0243; *p* for trend 0.0243), respectively).

**TABLE 3:** Associations between TyG index and mortalities of in‐hospital and 1‐year in crude and adjusted models.

Exposure	Crude model HR (95% CI), *p*‐value	Adjusted model A HR (95% CI), *p*‐value	Adjusted model B HR (95% CI), *p*‐value
In‐hospital mortality			
TyG index	0.79 (0.69, 0.92) 0.0019	0.81 (0.70, 0.94) 0.0049	0.79 (0.66, 0.94) 0.0086
TyG index (tertiles)			
T1 (<9.47)	Ref.	Ref.	Ref.
T2 (9.47–10.16)	0.86 (0.64, 1.15) 0.3066	0.87 (0.65, 1.17) 0.3636	0.85 (0.60, 1.21) 0.3753
T3 (>10.16)	0.65 (0.48, 0.88) 0.0049	0.67 (0.50, 0.91) 0.0112	0.67 (0.46, 0.96) 0.0314
*p* for trend	0.0049	0.0114	0.0315
TyG index (quartiles)			
Q1 (<9.27)	Ref.	Ref.	Ref.
Q2 (9.27–9.77)	0.68 (0.48, 0.96) 0.0267	0.67 (0.48, 0.95) 0.0244	0.57 (0.38, 0.87) 0.0088
Q3 (9.78–10.39)	0.70 (0.50, 0.98) 0.0381	0.71 (0.51, 1.01) 0.0551	0.69 (0.46, 1.04) 0.0788
Q4 (>10.39)	0.57 (0.41, 0.81) 0.0017	0.60 (0.42, 0.85) 0.0040	0.53 (0.34, 0.81) 0.0032
*p* for trend	0.0032	0.0078	0.0116
1‐year mortality			
TyG index	0.80 (0.69, 0.92) 0.0016	0.82 (0.71, 0.94) 0.0054	0.79 (0.66, 0.94) 0.0080
TyG index (tertiles)			
T1 (<9.47)	Ref.	Ref.	Ref.
T2 (9.47–10.16)	0.82 (0.62, 1.10) 0.1932	0.84 (0.63, 1.13) 0.2529	0.81 (0.57, 1.15) 0.2338
T3 (>10.16)	0.65 (0.49, 0.88) 0.0046	0.69 (0.51, 0.93) 0.0141	0.66 (0.46, 0.95) 0.0243
*p* for trend	0.0047	0.0141	0.0243
TyG index (quartiles)			
Q1 (<9.27)	Ref.	Ref.	Ref.
Q2 (9.27–9.77)	0.71 (0.50, 0.99) 0.0440	0.70 (0.50, 0.99) 0.0421	0.59 (0.39, 0.88) 0.0104
Q3 (9.78–10.39)	0.74 (0.53, 1.03) 0.0729	0.76 (0.54, 1.07) 0.1150	0.74 (0.49, 1.12) 0.1568
Q4 (>10.39)	0.61 (0.44, 0.86) 0.0047	0.65 (0.46, 0.91) 0.0130	0.58 (0.38, 0.88) 0.0107
*p* for trend	0.0087	0.0257	0.0413

*Note*: Crude model adjusted for: None; Model A adjusted for: age; gender; Model B adjusted for: age; gender; HR; DBP; SBP; RR; ALT; AG; Albumin; AST; bicarbonate; total bilirubin; total calcium; chloride; creatinine; haematocrit; haemoglobin; INR; lactate; PLT; PT; PTT; RDW; RBC; sodium; urea nitrogen; WBC; renal disease; CAD; hypertension; APAHCEII; SOFA.

Abbreviations: AG, anion gap; ALT, alanine aminotransferase; APACHE, acute physiology and chronic health evaluation; AST, aspartate aminotransferase; CAD, coronary artery disease; CI, confidential interval; DBP, diastolic blood pressure; HR, hazard ratio; HR, heart rate; INR, international normalized ratio; PLT, platelet; PT, prothrombin time; PTT, partial thrombin time; RBC, red blood cells; RDW, red blood cell distribution width; RR, respiratory rate; SBP, systolic blood pressure; SOFA, sequential organ failure assessment; TyG index, triglyceride glucose index; WBC, white blood cells.

In addition, we divided the cohort into four categorical groups based on the quartiles of TyG index: Q1 (<9.27), Q2 (9.27–9.77), Q3 (9.78–10.39) and Q4 (>10.39). Compared to Q1 group, the risk of mortalities of in‐hospital and 1‐year in Q4 group were also significantly lowest (HR = 0.53 (95% CI: 0.34–0.81, *p* = 0.0032; *p* for trend 0.0166) and HR = 0.58 (95% CI: 0.38–0.88, *p* = 0.0107; *p* for trend 0.0413), respectively).

**FIGURE 1:**
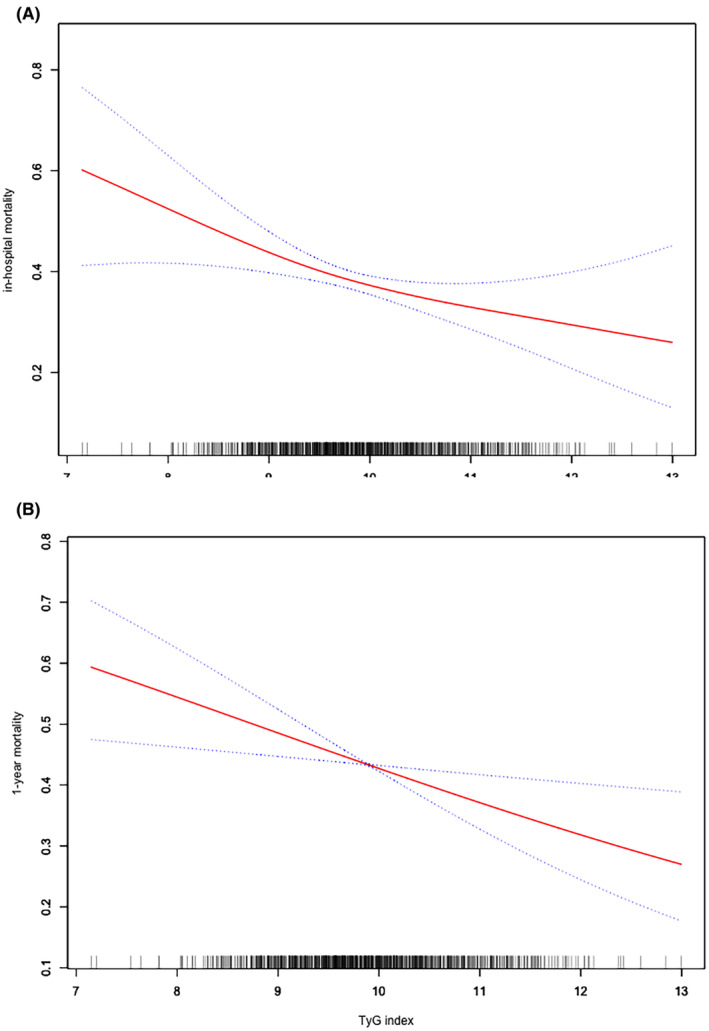
Smooth fitting curves demonstrated the associations between TyG index and mortalities of in‐hospital (A) and 1‐year (B).

**FIGURE 2:**
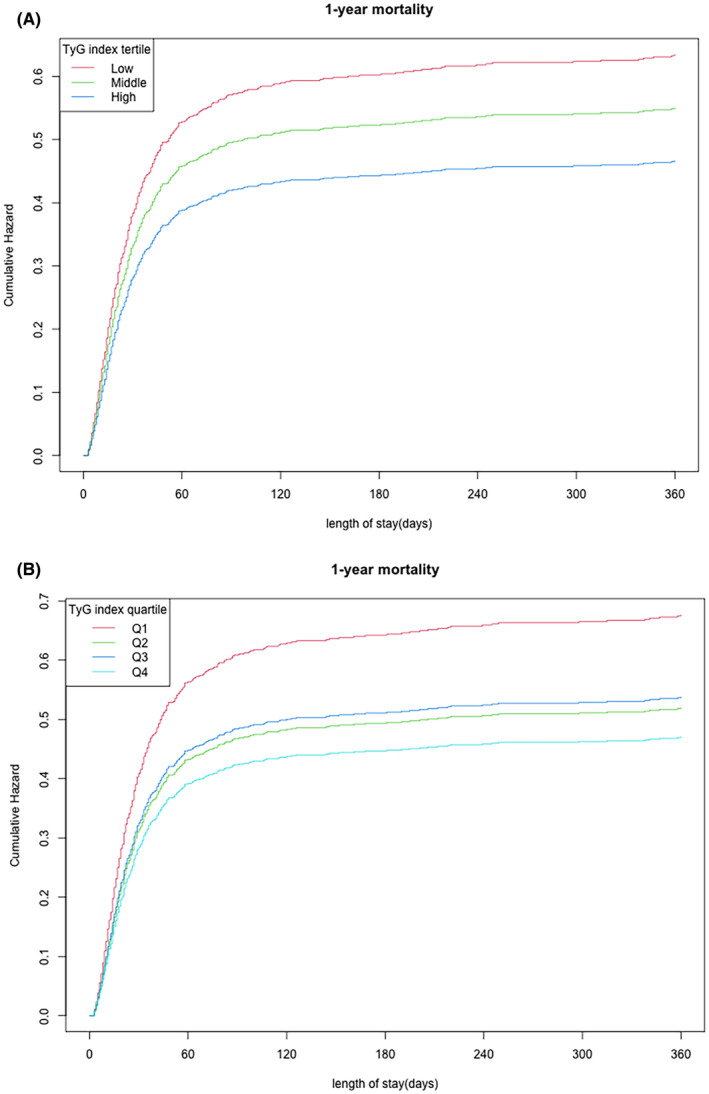
Kaplan–Meier analysis for cumulative hazard of 1‐year mortality in sepsis based on tertiles (A) and quartiles (B) of TyG index.

The smoothing curves were performed for illuminating the relationship between TyG index and mortalities of in‐hospital (Figure 1A) and 1‐year (Figure 1B). The Kaplan–Meier analysis for cumulative hazard of 1‐year mortality indicated there were significant differences between different TyG index groups: T1‐T3 groups (Figure 2A) and Q1–Q4 groups (Figure 2B). In the groups with higher levels of TyG index (T3 and Q4 groups), the cumulative hazards of 1‐year mortality in sepsis were the lowest (both *p* < 0.05).

### 3.5 Comparison of the linear model and the non‐linear model

We compared the linear model (model I) and non‐linear model (model II) in Table 4. For in‐hospital mortality, the linear model was better (*p* for the log‐likelihood ratio test = 0.080). When TyG index ≤9.33, the risk of in‐hospital mortality significantly decreased with the increment in TyG index (HR = 0.49, 95% CI: 0.27–0.87, *p* = 0.0145). When TyG index >**9.33**, there was also a negative relationship, but without statistically significant (HR = 0.91, 95% CI: 0.72–1.15, *p* = 0.4259). For 1‐year mortality, the linear model was also better (*p* for the log‐likelihood ratio test = 0.117). When TyG index ≤9.32, the risk of 1‐year mortality significantly decreased with the increment in TyG index (HR = 0.51, 95% CI: 0.28–0.92, *p* = 0.0243). When TyG index >9.32, there was also a negative relationship, but without statistically significant (HR = 0.89, 95% CI: 0.71–1.12, *p* = 0.3307).

**TABLE 4:** Analysis of the linear and non‐linear models for the relationship between TyG index and mortalities of in‐hospital and 1‐year in sepsis.

	Number (%)	HR(95% CI), *p*‐value
In‐hospital mortality		
Model I: The linear model	1103 (100%)	0.79 (0.66, 0.94) 0.0086
Model II: The non‐linear model		
The turning point of TyG index		9.33
≤9.33 (slope A)	294 (26.65%)	0.49 (0.27, 0.87) 0.0145
>9.33 (slope B)	809 (73.35%)	0.91 (0.72, 1.15) 0.4259
Slope B to slope A		1.87 (0.92, 3.78) 0.0833
Predicted at 9.33		−0.45 (−0.67, −0.22)
*p* for the log‐likelihood ratio test		0.080
1‐year mortality		
Model I: The linear model	1103 (100%)	0.79 (0.66, 0.94) 0.0080
Model II: The non‐linear model		
The turning point of TyG index		9.32
≤9.32 (slope A)	290 (26.29%)	0.51 (0.28, 0.92) 0.0243
>9.32 (slope B)	813 (73.71%)	0.89 (0.71, 1.12) 0.3307
Slope B to slope A		1.76 (0.86, 3.58) 0.1215
Predicted at 9.32		−0.24 (−0.46, −0.02)
*p* for the log‐likelihood ratio test		0.117

*Note*: Model I and II adjusted for: age; gender; HR; DBP; SBP; RR; ALT; AG; Albumin; AST; bicarbonate; total bilirubin; total calcium; chloride; creatinine; haematocrit; haemoglobin; INR; lactate; PLT; PT; PTT; RDW; RBC; sodium; urea nitrogen; WBC; renal disease; CAD; hypertension; APAHCEII; SOFA.

Abbreviations: AG, anion gap; ALT, alanine aminotransferase; APACHE, acute physiology and chronic health evaluation; AST, aspartate aminotransferase; CAD, coronary artery disease; CI, confidential interval; DBP, diastolic blood pressure; HR, hazard ratio; HR, heart rate; INR, international normalized ratio; PLT, platelet; PT, prothrombin time; PTT, partial thrombin time; RBC, red blood cells; RDW, red blood cell distribution width; RR, respiratory rate; SBP, systolic blood pressure; SOFA, sequential organ failure assessment; TyG index, triglyceride glucose index; WBC, white blood cells.

### 3.6 Subgroup analysis

In Table S2, we also constructed the subgroup analysis. Chloride, **RBC** and sodium were found to be interacted with the relationship between TyG index and in‐hospital mortality (*p* for interaction: 0.0207, 0.0217 and 0.0321, respectively). Chloride and sodium were found to be interacted with the relationship between TyG index and 1‐year mortality (*p* for interaction: 0.0346 and 0.0368, respectively).

## 4 DISCUSSION

In the present study, some points have been addressed. First, the negative relationship between TyG index and clinical outcomes in sepsis was found. Second, with the per unit increment in TyG index, the risk of in‐hospital and 1‐year mortality in sepsis both decreased by 21%.

We found a negative association between TyG index and mortalities of in‐hospital and 1‐year in sepsis. It might be inconsistent with previous studies which have done in some other diseases. The general characteristic of the sepsis cohort could partly explain the results. In our study, patients in non‐survivor group had lower levels of TyG index (Table 1). Non‐survivor group patients were older with higher levels of variables including total bilirubin, total calcium, PT, PTT, lactate and lower levels of chloride, haematocrit and haemoglobin. In addition, the incidence of septic shock and AKI were higher in non‐survivor group.

Since the TyG index was combined with TG and FBG, some explanations about the negative association between TyG index and outcomes in sepsis could be made on the both sides of TG and FBG. Disorders of metabolism in TG and FBG have been established risky factors for various diseases.^15^, ^16^, ^17^

The prognostic value of TG in sepsis has been seldom discussed yet, and some researches found that lower levels of TG were associated with higher risk of poor prognosis in neurocardiac diseases. One study in myocardial infarction revealed that TG < 110 mg/dL was confirmed as the cut‐off value for predicting 30‐day mortality (HR = 5.05, 95% CI: 1.75–14.54).^18^ Moreover, decreased level of TG was an indicator of recurrent ischemia in myocardial infarction.^19^ In heart failure patients, with TG levels decreasing, the risk of cardiac death increased.^20^ One prospective cohort study among 27,937 female participants with a median of 19.3 years' follow‐up concluded that participants with the lowest quartile levels of TG had the highest morbidity of hemorrhagic stroke compared with those participants in the top quartile of TG.^21^

Lower levels of FBG have been found to be associated with outcomes in sepsis and non‐sepsis diseases. A systemic review including more than one hundred prospective researches with more than 2 million participants clarified that when the level of FBG less than 4.0 mmol/L, the risks of stroke, cardiovascular events and all‐cause mortality increased significantly (HR = 1.08, 95% CI: 1.03–1.13; HR = 1.05, 95% CI: 1.03–1.07; HR = 1.56, 95% CI: 1.09–2.23; respectively).^22^ In sepsis, compared to the patients with normal FBG, the HR for short‐term mortality in patients with mild hypoglycemia and severe hypoglycemia were 7.56 (95% CI: 2.96–19.35) and 8.18 (95% CI: 2.39–27.96), respectively.^23^ Moreover, mild hypoglycemia was significantly related with a higher 1‐year cumulative mortality rate in sepsis patients.^24^

Based on the previous related researches, the possible mechanism of the negative relationship between TyG index and prognosis in sepsis could be illuminated as follow. First, TG is a significant energy source for tissues and organs. Lower TyG index might be correlated to a worse nutrition status, which could contribute to the worsening of sepsis.^25^ Second, evidences proved that there was a close relationship between sympathetic activity and the regulation and metabolism of TG.^19^ Decreased plasma TG in the early stage of sepsis could be an indicator of enhanced sympathetic activity, which in turn may increase the risk of poor prognosis. Third, hypoglycemia might be involved with various pathophysiological effects in sepsis patients including enhanced systemic inflammatory responses, impairment of the corticosteroid response, induction of unstable hemodynamics and cardiac arrythmia, which result in worse outcomes.^26^, ^27^, ^28^, ^29^

The strength of our study was that we found a negative relationship between TyG index and clinical outcomes in sepsis patients. However, our study had some limitations. First, the relationship between TyG index and outcomes in sepsis was not cause‐effect. Second, we only used the one time of TyG index and didn't dynamically evaluate the TyG index. TyG index might be affected by medications and progression of sepsis. Third, it was a retrospective study based on a public database, so we couldn't rule out all confounding factors.

## 5 CONCLUSION

In the study, a negative relationship between TyG index and outcomes of short‐term and long‐term in sepsis was found.

## AUTHOR CONTRIBUTIONS

**Yan Cao:** Funding acquisition (equal); methodology (equal); resources (equal). **Liudang He:** Software (equal); supervision (equal). **Yingjie Su:** Funding acquisition (equal); methodology (equal). **Ju Luo:** Validation (equal); visualization (equal); writing – original draft (equal). **Ning Ding:** Conceptualization (equal); writing – original draft (equal); writing – review and editing (equal).

## FUNDING INFORMATION

National natural science foundation of China (No. 82372178), Changsha Natural Science Foundation (kq2208445), Changsha Central Hospital (YNKY202306), Changsha Science and Technology Bureau (kzd21084, kzd22074, kzd22075), National Key Clinical Specialty Scientific Research Project (Z2023047).

## CONFLICT OF INTEREST STATEMENT

The authors have no conflict of interest to declare.

## Supporting information


**Figure S1:** Flow chart for patient’s enrollment and study design.

**Table S1:** Description of the study cohort.

**Table S2:** Subgroups and interaction analyses.

## Data Availability

The data that support the findings of this study are available from the Massachusetts Institute of Technology (MIT) and Beth Israel Deaconess Medical Center (BIDMC) but restrictions apply to the availability of these data, which were used under licence for the current study, and so are not publicly available. Data are however available from the authors upon reasonable request and with permission of the Massachusetts Institute of Technology (MIT) and Beth Israel Deaconess Medical Center (BIDMC).
