# Supplementary material for: Triglyceride‐glucose index and clinical outcomes in sepsis: A retrospective cohort study of MIMIC‐IV
Source: J Cell Mol Med. 2024 Aug 28;28(16):e70007. doi: 10.1111/jcmm.70007 (PMC11358033; doi:10.1111/jcmm.70007)
Supplement: Supplementary file 1 — Figure S1: Flow chart for patient’s enrollment and study design. [file JCMM-28-e70007-s002.pdf]

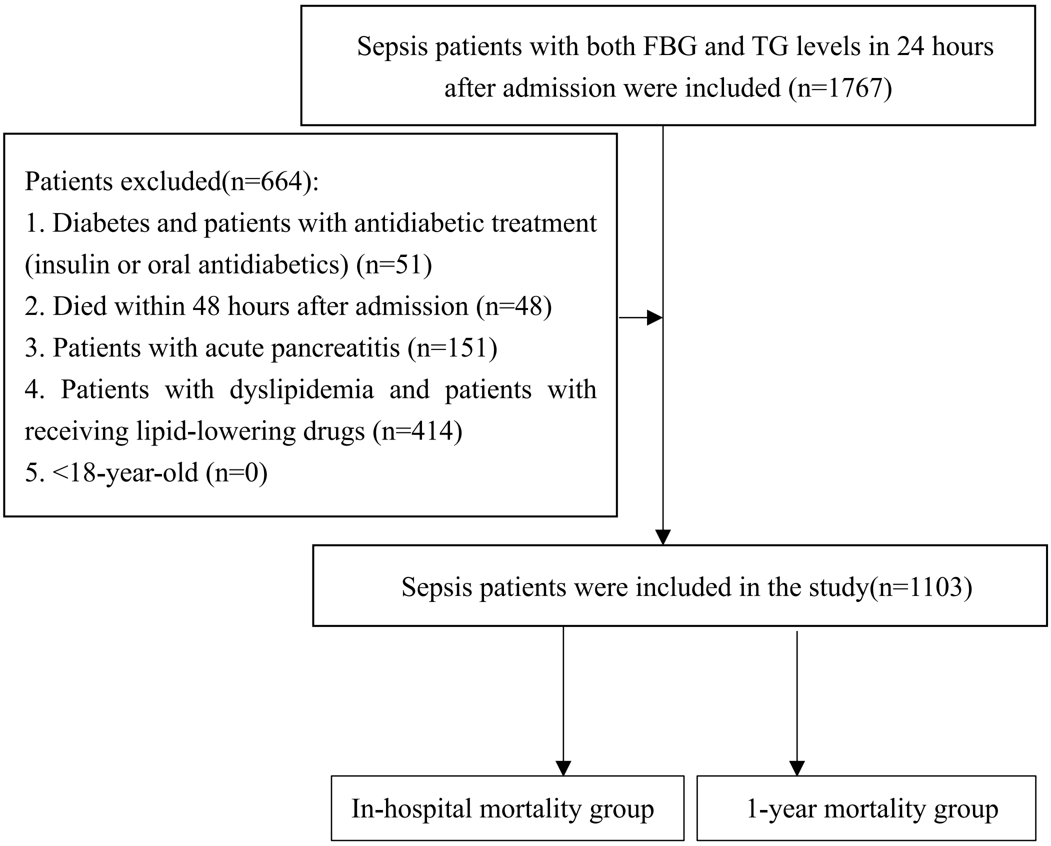

Supplementary Figure 1: Flow chart for patients enrollment and study design.
Abbreviations: FBG=fasting blood sugar, TG=triglyceride
