# Supplementary material for: Triglyceride‐glucose index and clinical outcomes in sepsis: A retrospective cohort study of MIMIC‐IV
Source: J Cell Mol Med. 2024 Aug 28;28(16):e70007. doi: 10.1111/jcmm.70007 (PMC11358033; doi:10.1111/jcmm.70007)
Supplement: Supplementary file 2 — Table S1: Description of the study cohort. [file JCMM-28-e70007-s001.docx]

**Supplementary Table 1 Description of the study cohort**

| **Characteristics** | **Sepsis patients** |
| --- | --- |
| Number | 1103 |
| Age(years) (median, IQR) | 61.00 (50.00-71.00) |
| **Gender** (n,%) |  |
| Male | 642 (58.20%) |
| Female | 461 (41.80%) |
| **Marital status**(n,%) |  |
| Married | 465 (42.16%) |
| Singled | 347 (31.46%) |
| Divorced | 79 (7.16%) |
| Others | 212 (19.22%) |
| **Ethnicity**(n,%) |  |
| White | 712 (64.55%) |
| Black/African American | 101 (9.16%) |
| Asian | 32 (2.90%) |
| Hispanic/Latino | 43 (3.90%) |
| Others | 215 (19.49%) |
| **Managements**(n,%) |  |
| RRT | 255 (23.12%) |
| Ventilator use | 893 (80.96%) |
| Vasopressor use | 355 (32.18%) |
| **Organ dysfunction(**n,%**)**  AKI | 853(77.33%) |
| Septic shock | 493 (44.70%) |
| **Clinical outcomes** |  |
| In-hospital mortality(n,%) | 414 (37.53%) |
| 1-year mortality(n,%) | 466 (42.25%) |

**Abbreviations:** RRT=renal replacement therapy, AKI=acute kidney injury.
