# Supplementary material for: Triglyceride‐glucose index and clinical outcomes in sepsis: A retrospective cohort study of MIMIC‐IV
Source: J Cell Mol Med. 2024 Aug 28;28(16):e70007. doi: 10.1111/jcmm.70007 (PMC11358033; doi:10.1111/jcmm.70007)
Supplement: Supplementary file 3 — Table S2: Subgroups and interaction analyses. [file JCMM-28-e70007-s003.docx]

**Supplementary Table 2: Subgroups and interaction analyses**

|  | **In-hospital mortality** | P for interaction | **1-year mortality** | P for interaction |
| --- | --- | --- | --- | --- |
| Variables | HR (95%CI), P-value |  | HR (95%CI), P-value |  |
| Gender |  | 0.3566 |  | 0.3228 |
| Male | 0.84 (0.70, 1.01) 0.0607 |  | 0.84 (0.70, 1.01) 0.0593 |  |
| Female | 0.73 (0.58, 0.92) 0.0085 |  | 0.73 (0.58, 0.92) 0.0069 |  |
| Age |  | 0.9809 |  | 0.5329 |
| Low | 0.80 (0.62, 1.04) 0.0980 |  | 0.74 (0.57, 0.96) 0.0228 |  |
| Middle | 0.81 (0.63, 1.04) 0.0933 |  | 0.80 (0.63, 1.03) 0.0808 |  |
| High | 0.83 (0.65, 1.06) 0.1362 |  | 0.91 (0.71, 1.15) 0.4241 |  |
| Renal disease |  | 0.6269 |  | 0.6251 |
| No | 0.80 (0.69, 0.93) 0.0031 |  | 0.80 (0.69, 0.93) 0.0026 |  |
| Yes | 0.65 (0.28, 1.51) 0.3147 |  | 0.65 (0.28, 1.51) 0.3147 |  |
| CAD |  | 0.5508 |  | 0.5217 |
| No | 0.80 (0.69, 0.93) 0.0040 |  | 0.81 (0.70, 0.93) 0.0036 |  |
| Yes | 0.66 (0.36, 1.23) 0.1941 |  | 0.66 (0.35, 1.22) 0.1808 |  |
| Hypertension |  | 0.7439 |  | 0.8541 |
| No | 0.80 (0.68, 0.94) 0.0076 |  | 0.80 (0.68, 0.94) 0.0056 |  |
| Yes | 0.75 (0.54, 1.06) 0.1033 |  | 0.77 (0.56, 1.08) 0.1268 |  |
| HR |  | 0.3065 |  | 0.5027 |
| Low | 0.67 (0.51, 0.89) 0.0057 |  | 0.70 (0.54, 0.93) 0.0118 |  |
| Middle | 0.75 (0.59, 0.97) 0.0269 |  | 0.75 (0.59, 0.96) 0.0212 |  |
| High | 0.90 (0.70, 1.15) 0.3965 |  | 0.87 (0.68, 1.11) 0.2653 |  |
| DBP |  | 0.9838 |  | 0.6730 |
| Low | 0.78 (0.60, 1.01) 0.0613 |  | 0.75 (0.58, 0.97) 0.0285 |  |
| Middle | 0.76 (0.58, 0.99) 0.0396 |  | 0.73 (0.57, 0.94) 0.0164 |  |
| High | 0.78 (0.61, 1.01) 0.0577 |  | 0.85 (0.66, 1.08) 0.1875 |  |
| SBP |  | 0.4760 |  | 0.3711 |
| Low | 0.68 (0.52, 0.89) 0.0046 |  | 0.67 (0.51, 0.87) 0.0029 |  |
| Middle | 0.81 (0.63, 1.05) 0.1065 |  | 0.81 (0.63, 1.03) 0.0898 |  |
| High | 0.84 (0.65, 1.08) 0.1788 |  | 0.86 (0.67, 1.11) 0.2464 |  |
| RR |  | 0.4948 |  | 0.5926 |
| Low | 0.68 (0.51, 0.89) 0.0046 |  | 0.68 (0.53, 0.89) 0.0052 |  |
| Middle | 0.84 (0.65, 1.09) 0.1989 |  | 0.82 (0.64, 1.06) 0.1295 |  |
| High | 0.79 (0.62, 1.01) 0.0647 |  | 0.79 (0.62, 1.01) 0.0622 |  |
| ALT |  | 0.7479 |  | 0.6968 |
| Low | 0.84 (0.64, 1.09) 0.1785 |  | 0.84 (0.65, 1.08) 0.1754 |  |
| Middle | 0.76 (0.59, 0.97) 0.0309 |  | 0.78 (0.61, 1.00) 0.0532 |  |
| High | 0.73 (0.56, 0.94) 0.0164 |  | 0.72 (0.56, 0.93) 0.0116 |  |
| AG |  | 0.0570 |  | 0.1483 |
| Low | 0.67 (0.51, 0.89) 0.0061 |  | 0.67 (0.51, 0.88) 0.0038 |  |
| Middle | 1.04 (0.79, 1.36) 0.8023 |  | 0.97 (0.74, 1.27) 0.8265 |  |
| High | 0.72 (0.57, 0.90) 0.0039 |  | 0.77 (0.62, 0.95) 0.0164 |  |
| AST |  | 0.6458 |  | 0.4417 |
| Low | 0.83 (0.64, 1.08) 0.1685 |  | 0.85 (0.66, 1.10) 0.2243 |  |
| Middle | 0.78 (0.60, 1.01) 0.0599 |  | 0.80 (0.62, 1.02) 0.0761 |  |
| High | 0.70 (0.55, 0.90) 0.0048 |  | 0.68 (0.53, 0.87) 0.0022 |  |
| Albumin |  | 0.6570 |  | 0.8115 |
| Low | 0.70 (0.54, 0.92) 0.0093 |  | 0.72 (0.56, 0.93) 0.0133 |  |
| Middle | 0.78 (0.61, 1.00) 0.0504 |  | 0.80 (0.63, 1.02) 0.0692 |  |
| High | 0.84 (0.64, 1.10) 0.2015 |  | 0.80 (0.61, 1.05) 0.1022 |  |
| Bicarbonate |  | 0.8864 |  | 0.7666 |
| Low | 0.76 (0.59, 0.97) 0.0278 |  | 0.74 (0.59, 0.95) 0.0158 |  |
| Middle | 0.81 (0.63, 1.04) 0.1055 |  | 0.84 (0.66, 1.07) 0.1643 |  |
| High | 0.83 (0.64, 1.07) 0.1487 |  | 0.82 (0.63, 1.06) 0.1225 |  |
| Total bilirubin |  | 0.2031 |  | 0.3657 |
| Low | 0.67 (0.50, 0.91) 0.0097 |  | 0.69 (0.52, 0.92) 0.0128 |  |
| Middle | 0.94 (0.73, 1.21) 0.6319 |  | 0.90 (0.70, 1.15) 0.4086 |  |
| High | 0.74 (0.58, 0.94) 0.0125 |  | 0.76 (0.60, 0.96) 0.0206 |  |
| Total calcium |  | 0.8735 |  | 0.8365 |
| Low | 0.78 (0.60, 1.01) 0.0604 |  | 0.78 (0.60, 1.00) 0.0496 |  |
| Middle | 0.79 (0.61, 1.03) 0.0768 |  | 0.79 (0.61, 1.02) 0.0675 |  |
| High | 0.85 (0.67, 1.08) 0.1873 |  | 0.86 (0.68, 1.09) 0.2065 |  |
| Chloride |  | 0.0207 |  | 0.0346 |
| Low | 0.75 (0.58, 0.96) 0.0202 |  | 0.76 (0.59, 0.96) 0.0233 |  |
| Middle | 0.62 (0.47, 0.81) 0.0005 |  | 0.63 (0.49, 0.82) 0.0007 |  |
| High | 1.03 (0.80, 1.32) 0.8090 |  | 1.01 (0.79, 1.28) 0.9652 |  |
| Creatinine |  | 0.2043 |  | 0.2133 |
| Low | 0.86 (0.63, 1.16) 0.3159 |  | 0.86 (0.64, 1.16) 0.3218 |  |
| Middle | 0.87 (0.69, 1.11) 0.2727 |  | 0.86 (0.68, 1.09) 0.2156 |  |
| High | 0.66 (0.52, 0.83) 0.0005 |  | 0.66 (0.53, 0.83) 0.0004 |  |
| Hematocrit |  | 0.0846 |  | 0.3459 |
| Low | 0.99 (0.78, 1.25) 0.9277 |  | 0.93 (0.74, 1.18) 0.5606 |  |
| Middle | 0.67 (0.51, 0.88) 0.0035 |  | 0.75 (0.58, 0.97) 0.0298 |  |
| High | 0.77 (0.59, 1.00) 0.0500 |  | 0.75 (0.57, 0.97) 0.0276 |  |
| Hemoglobin |  | 0.0999 |  | 0.2808 |
| Low | 0.99 (0.78, 1.26) 0.9453 |  | 0.94 (0.74, 1.19) 0.6120 |  |
| Middle | 0.70 (0.54, 0.91) 0.0069 |  | 0.77 (0.60, 0.99) 0.0421 |  |
| High | 0.73 (0.56, 0.96) 0.0251 |  | 0.71 (0.55, 0.93) 0.0135 |  |
| INR |  | 0.3046 |  | 0.4321 |
| Low | 0.86 (0.68, 1.10) 0.2356 |  | 0.88 (0.69, 1.12) 0.2915 |  |
| Middle | 0.92 (0.70, 1.22) 0.5856 |  | 0.87 (0.66, 1.13) 0.3020 |  |
| High | 0.70 (0.55, 0.90) 0.0056 |  | 0.72 (0.56, 0.92) 0.0081 |  |
| Lactate |  | 0.3272 |  | 0.0863 |
| Low | 0.88 (0.67, 1.15) 0.3497 |  | 0.97 (0.75, 1.25) 0.8003 |  |
| Middle | 0.67 (0.52, 0.87) 0.0026 |  | 0.64 (0.49, 0.83) 0.0008 |  |
| High | 0.81 (0.64, 1.03) 0.0847 |  | 0.80 (0.63, 1.00) 0.0532 |  |
| PLT |  | 0.8252 |  | 0.8624 |
| Low | 0.83 (0.66, 1.05) 0.1240 |  | 0.84 (0.66, 1.05) 0.1289 |  |
| Middle | 0.81 (0.63, 1.04) 0.0975 |  | 0.79 (0.62, 1.01) 0.0645 |  |
| High | 0.74 (0.56, 0.98) 0.0360 |  | 0.76 (0.58, 0.99) 0.0422 |  |
| PT |  | 0.0620 |  | 0.1162 |
| Low | 0.89 (0.70, 1.14) 0.3696 |  | 0.88 (0.69, 1.13) 0.3167 |  |
| Middle | 0.99 (0.76, 1.29) 0.9343 |  | 0.95 (0.74, 1.22) 0.6790 |  |
| High | 0.65 (0.50, 0.84) 0.0012 |  | 0.66 (0.51, 0.86) 0.0020 |  |
| PTT |  | 0.6444 |  | 0.6876 |
| Low | 0.93 (0.71, 1.22) 0.5992 |  | 0.92 (0.71, 1.18) 0.5011 |  |
| Middle | 0.86 (0.66, 1.12) 0.2651 |  | 0.86 (0.66, 1.11) 0.2414 |  |
| High | 0.78 (0.61, 1.00) 0.0532 |  | 0.78 (0.61, 1.00) 0.0520 |  |
| RDW |  | 0.7469 |  | 0.3954 |
| Low | 0.87 (0.65, 1.15) 0.3130 |  | 0.94 (0.72, 1.23) 0.6594 |  |
| Middle | 0.77 (0.59, 1.00) 0.0541 |  | 0.73 (0.56, 0.95) 0.0183 |  |
| High | 0.87 (0.69, 1.10) 0.2574 |  | 0.87 (0.69, 1.10) 0.2312 |  |
| RBC |  | 0.0217 |  | 0.0595 |
| Low | 1.02 (0.81, 1.28) 0.8798 |  | 1.00 (0.79, 1.26) 0.9933 |  |
| Middle | 0.62 (0.48, 0.81) 0.0005 |  | 0.66 (0.51, 0.85) 0.0014 |  |
| High | 0.85 (0.65, 1.13) 0.2701 |  | 0.84 (0.64, 1.10) 0.2057 |  |
| Sodium |  | 0.0321 |  | 0.0368 |
| Low | 0.61 (0.47, 0.80) 0.0004 |  | 0.62 (0.48, 0.81) 0.0004 |  |
| Middle | 0.80 (0.63, 1.02) 0.0699 |  | 0.80 (0.63, 1.01) 0.0580 |  |
| High | 1.00 (0.78, 1.30) 0.9832 |  | 0.99 (0.77, 1.28) 0.9646 |  |
| Urea nitrogen |  | 0.4211 |  | 0.4522 |
| Low | 0.91 (0.69, 1.19) 0.4797 |  | 0.86 (0.65, 1.13) 0.2733 |  |
| Middle | 0.70 (0.55, 0.91) 0.0069 |  | 0.69 (0.54, 0.88) 0.0035 |  |
| High | 0.78 (0.62, 0.98) 0.0351 |  | 0.82 (0.65, 1.02) 0.0803 |  |
| WBC |  | 0.0705 |  | 0.0674 |
| Low | 0.95 (0.74, 1.24) 0.7198 |  | 0.93 (0.72, 1.19) 0.5512 |  |
| Middle | 0.86 (0.67, 1.11) 0.2583 |  | 0.89 (0.70, 1.14) 0.3648 |  |
| High | 0.64 (0.50, 0.82) 0.0004 |  | 0.64 (0.50, 0.81) 0.0003 |  |

**Abbreviations:** CAD= coronary artery disease, SBP=systolic blood pressure, DBP= diastolic blood pressure, HR= heart rate, RR=respiratory rate, ALT=alanine aminotransferase, AG=anion gap, AST= aspartate aminotransferase, INR= international normalized ratio, PLT=platelet, PT= prothrombin time, PTT=partial thrombin time, RDW=red blood cell distribution width, RBC=red blood cells, WBC=white blood cells, HR=hazard ratio, CI=confidential interval.
